# Clinicopathological findings, treatment, and outcome in 60 cats with gastrointestinal eosinophilic sclerosing fibroplasia

**DOI:** 10.1111/jvim.16992

**Published:** 2024-01-11

**Authors:** Petra Černá, Cristobal Lopez‐Jimenez, Kenjiro Fukushima, Ko Nakashima, Taisuke Nakagawa, Fiona Adam, Anna Groth, Andrew Denning, Nicolas Israeliantz, Danièlle A. Gunn‐Moore

**Affiliations:** ^1^ Department of Clinical Sciences Colorado State University Fort Collins Colorado USA; ^2^ Small Animal Clinic The University of Veterinary Sciences Brno Brno Czech Republic; ^3^ North Downs Specialist Referrals Bletchingley UK; ^4^ Veterinary Specialists & Emergency Center Kawaguchi‐shi Saitama Japan; ^5^ Small Animal Medical Center Saitama Japan; ^6^ Veterinary Medical Centre The University of Tokyo Tokyo Japan; ^7^ The Royal (Dick) School of Veterinary Studies University of Edinburgh Midlothian UK

**Keywords:** eosinophilia, gastrointestinal mass, mesenteric mass, ragdoll

## Abstract

**Background:**

Gastrointestinal eosinophilic sclerosing fibroplasia (GESF) in cats presents as mass(es) associated with the gastrointestinal tract, mesentery, and abdominal lymph nodes.

**Hypothesis/Objectives:**

To report the clinicopathological findings, treatment, and outcome of cats with GESF.

**Animals:**

Sixty client‐owned cats diagnosed with GESF.

**Methods:**

Retrospective review of medical records of cats with histopathologically confirmed GESF.

**Results:**

The median age was 5.4 years (interquartile range [IQR], 3.3‐8.9.); 30% were Domestic Shorthairs and 12% were Domestic Longhair cats, with the most prevalent pedigree breeds being Ragdolls (25%), Exotic Shorthair (10%) and Persian (8%) cats. The median duration of clinical signs was 90 days (IQR, 17.5‐247.0); the most common clinical signs were weight loss (60%), hyporexia/anorexia (55%), chronic vomiting (37%), lethargy (35%) and chronic diarrhea (27%). Masses were located in the small intestine (32%), stomach (27%), ileocolic junction (15%), colon (10%), lymph node (8%) and mesentery (8%) and 15% of cats had >1 mass. Eosinophilia was present in 50% and hypoalbuminemia in 28% of cats. The mass was removed surgically in 37% of cases. Most cats (98%) were treated with corticosteroids. Survival was not statistically different between cats treated with surgical resection and cats treated with medical therapy alone, 88% of the cats were still alive at the time of writing.

**Conclusions and Clinical Importance:**

GESF is an important differential diagnosis for abdominal masses in cats, and has a much better prognosis than previously reported.

**Abbreviations**
FGESF: feline gastrointestinal eosinophilic sclerosing fibroplasia
FISH: fluorescence in situ hybridization
FNA: fine‐needle aspirates
PAS: periodic acid‐Schiff

## 1 INTRODUCTION

Gastrointestinal eosinophilic sclerosing fibroplasia (GESF) in cats is a recently described disease that presents as eosinophilic mass(es) in the gastrointestinal tract and associated abdominal lymph nodes, most commonly near the pylorus or ileocolic junction.^1^, ^2^ There are 2 case reports of GESF localized to the mesentery or retroperitoneum in cats.^3^, ^4^ A case report describes the same type of lesion outside of the abdominal cavity; eosinophilic sclerosing lymphadenitis in medial retropharyngeal lymph node was associated with *Pseudomonas aeruginosa* infection.^5^ Gastrointestinal eosinophilic sclerosing fibroplasia in cats is likely underdiagnosed because these mass lesions can be misinterpreted as lymphoma, granuloma, fibrosarcoma, adenocarcinoma, and mast cell tumor, and the histopathological diagnosis can be challenging.^2^, ^6^, ^7^, ^8^ Immunohistochemical staining for transforming growth factor β1 can aid diagnosis.^9^ This disease is most commonly seen in middle aged and male cats of all breeds, with Ragdolls being overrepresented and the disease is also reported in Maine Coons, Persians, Exotic Shorthairs, Bengal, and Scottish fold cats.^1^, ^2^, ^10^, ^11^, ^12^ The pathogenesis of GESF is still poorly understood; however, with some breeds, such as Ragdolls, being overrepresented, a genetic predisposition could be considered.

The most common presenting signs of cats with GESF are chronic vomiting, diarrhea, followed by weight loss, lethargy; less commonly an acute onset of vomiting and diarrhea is been reported.^1^, ^2^ A palpable intestinal mass is reported in 85% to 100% of cases, with abdominal pain and pyrexia being less common.^1^, ^2^ Surgical removal of the mass has been performed in most cases; however, several studies report medical management with administration of corticosteroids, cyclosporine, and mycophenolate.^2^, ^13^, ^14^, ^15^ A mass in a second location develops in some of the cases, after surgical removal of the initial mass.^16^, ^17^

The prognosis varies between studies; however, no large studies on prognosis or response to treatment have been reported.^2^ The objective of this study was to retrospectively evaluate a large number of cats with GESF, including their presentation, diagnosis, treatment, and outcome.

## 2 MATERIALS AND METHODS

### 2.1 Case recruitment

This is a retrospective, multicentric study, of cases of GESF in cats which have been collected by several veterinary hospitals around the world (USA, UK, and Japan) by contacting veterinarians that have previously seen cases of GESF in cats between 2010 and 2022. Inclusion criteria was confirmation of GESF diagnosis in cats by histopathology after surgical removal or biopsy of the mass; histopathology was performed by different pathologist from referral hospitals or referral laboratories. Cat signalment, clinical signs, physical findings, clinicopathological results, surgical reports and medical management were tabulated in an Excel (Microsoft, Redmond, WA, USA) spreadsheet. Ethical approval was gained from University of Edinburgh (VERC Reference: 17.22).

### 2.2 Statistical analysis

Survival times were measured from the date of presentation until the date of death or last follow‐up. Kaplan‐Meier analysis and log rank tests were used for survival analysis in order to evaluate the association of survival time with treatment (GraphPad Prism 9, GraphPad Software, Boston, MA, USA). Results were considered significant if *P* < .05.

## 3 RESULTS

### 3.1 Presentation and clinical signs

A total of 60 cats met the inclusion criteria for the study. The median age was 5.4 years (interquartile range [IQR], 3.3‐8.9). Of the 60 cats, 18 (30%) were Domestic Shorthair, 7 (12%) Domestic Longhair cats, and 35 (58%) were pedigree breeds: Ragdolls 15/60 (25%), Exotic Shorthair 6/60 (10%), Persian 5/60 (8%), Maine Coon 3/60 (5%), Sacred Birman 2/60 (3%), American Shorthair 1/60 (2%), Bengal 1/60 (2%), Bobtail 1/60 (2%), and British Shorthair 1/60 (2%). Of the 60 cats, 34 (57%) of the cats were neutered males, 25 (42%) were spayed females, and 1 (2%) entire female.

The median duration of clinical signs was 90 days (IQR, 17.5‐247.0) with most cats showing median of 3 (IQR, 2‐4) clinical signs. The most common clinical signs are reported in Table 1.

**TABLE 1:** Presenting clinical signs of cats with gastrointestinal eosinophilic sclerosing fibroplasia.

Clinical sign	Number of cats (%)
Weight loss	36/60 (60)
Hyporexia/anorexia	33/60 (55)
Chronic (>2 weeks) vomiting	22/60 (37)
Lethargy	21/60 (35)
Chronic diarrhea	16/60 (27)
Acute (<2 weeks) vomiting	8/60 (13)
Acute diarrhea	6/60 (10)
Constipation	6/60 (10)
Tenesmus	5/60 (8)
Polyphagia	4/60 (7)
Hematochezia	4/60 (7)
Decreased grooming	3/60 (5)
Melena	1/60 (2)
Excessive grooming	1/60 (2)

On physical examination, the most common abnormality was a palpable abdominal mass in 35 (58%) of the 60 cats, followed by pyrexia, in 9/60 (15%), dehydration, 7/60 (12%), and abdominal pain in 4/60 (7%). In 16 (26%) of the 60 cats, the body condition score was reported as less than ideal (<4/9).

### 3.2 Clinicopathological findings

Complete blood cell count findings were available for 57 (95%) of the 60 cats. The most common abnormalities were eosinophilia which was present in 30 (52%) of the 57 cases—the reference intervals varied between the clinics and the eosinophilia was mostly moderate to severe with the median percentage above RI 243.3 (IQR, 188.7‐465.2); however, not all medical records contained actual eosinophil numbers (some records only mentioned eosinophilia being present). Second most common hematological abnormality was anemia in 16 (28%). Less common findings included neutrophilia 10/57 (18%), monocytosis 7/57 (12%), lymphocytosis 5/57 (9%), basophilia 3/57 (5%), and neutropenia 1/57 (2%). Serum biochemistry findings were available for 58 (97%) of the 60 cats. The most common abnormality was hypoalbuminemia which was seen in 16/58 (28%) of cases. The reference intervals varied between the clinics and the hypoalbuminemia was mostly mild with the median percentage below RI 91.3 (IQR, 83.7‐96.2). The second most common abnormality was hyperglobulinemia seen in 8/58 (14%), followed by hypocholesterolemia in 6/58 (10%) and total hypocalcemia 6/58 (10%); with 4/6 (67%) hypocalcemic cats having normal albumin levels. Serum cobalamin was measured in 11 (18%) of the 60 cats and was normal in all of them, although it was at the low end of the reference interval in 1 cat (278; reference interval [RI], 214‐1106 ng/L). Folate measurement was available in 8/60 cats and was abnormally high in 5/8 cats.

### 3.3 Diagnostic imaging

**FIGURE 1:**
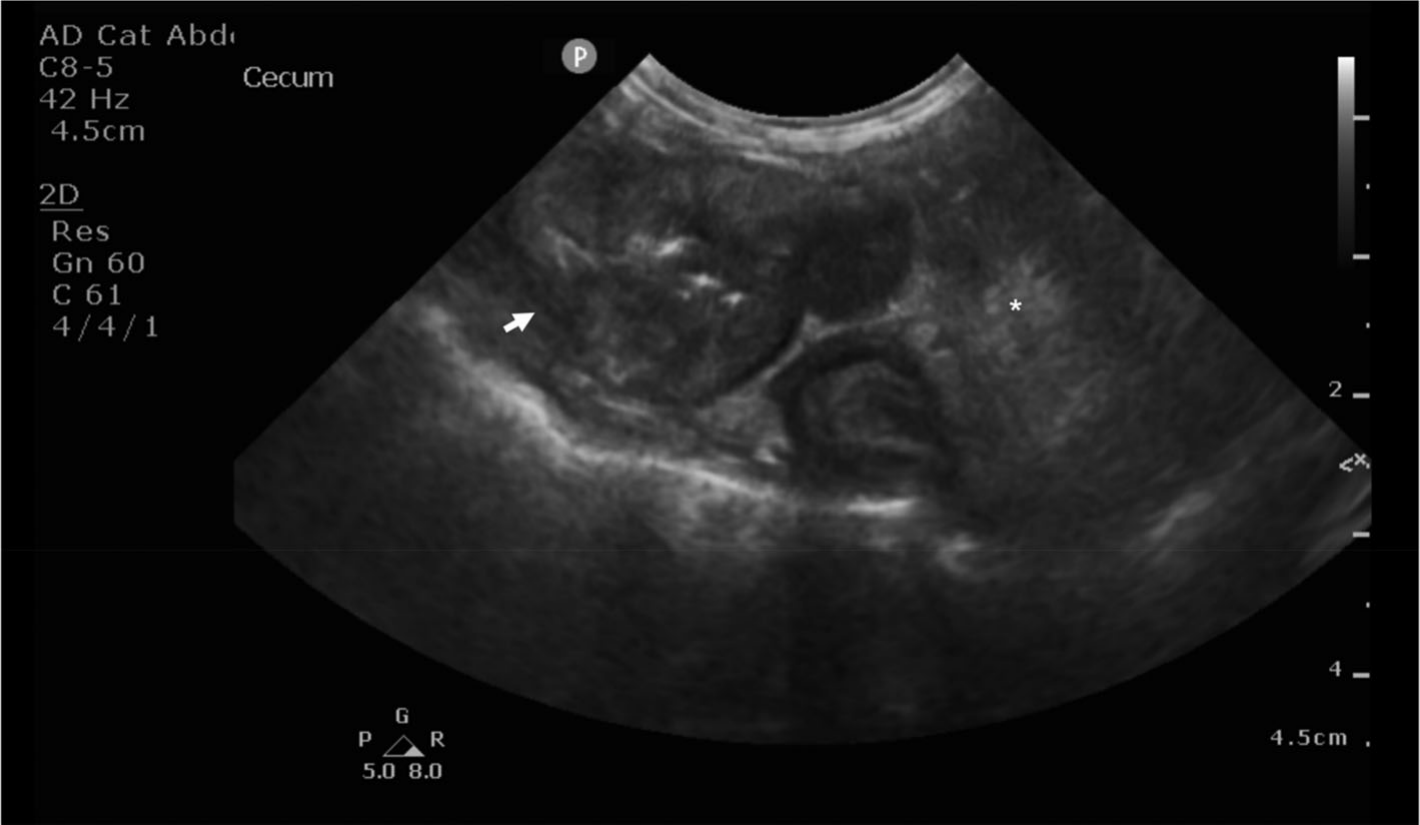
Ultrasonographic image of a mass at the level of the ileocolic junction (white arrow). The mass shows loss of layering, circumferential thickening and eccentric growth. The wall is heterogeneously mixed in echogenicity because of hyperechoic areas. The surrounding peritoneum is hyperechoic (asterisk).

Abdominal imaging was performed in all cats; however, ultrasound images were only available for 30 (50%) of the 60 cats; the others had abdominal radiographs performed and ultrasound reports was part of the medical records, but ultrasound images were not available for review. In 25/30 (83%) of the cats the mass originated from the stomach or intestines. Of the other 5 cases, 3 (10%) affected abdominal lymph nodes and 2 (7%) involved the mesentery (7%). The majority of gastrointestinal masses were associated with loss of the intestinal layering (Figure 1), symmetrical or asymmetrical circumferential thickening, eccentric growth and a heterogeneously mixed wall echogenicity which had hyperechoic areas and possible ulceration. In 6 (20%) of the 30 cases, these masses were reported to be associated with altered rather than lost layering. Hyperechoic areas were noted in 84% of the gastric or intestinal cases, and 80% of all cases, whereas thickening of the muscularis layer in the small intestine was seen in 33% of them. Peritoneal changes were reported in 22/30 (73%) of the cats, of which 19 (86%) had hyperechoic peritoneum and 8 (36%) had a peritoneal effusion (the amount of effusion was not always reported in the medical records). None of the lesions showed ultrasonographic findings compatible with gastrointestinal perforation. Enlarged lymph nodes were present in 27 (90%) of the 30 cases where ultrasound images were available; the most commonly affected lymph nodes were ileocolic in 9/27 (33%), followed by pancreaticoduodenal 8/27 (30%), and mesenteric, 8/27 (30%). For the rest of the cases (30 cats), abdominal ultrasound images were not available to assess the lymph nodes further.

### 3.4 Location of the mass

**FIGURE 2:**
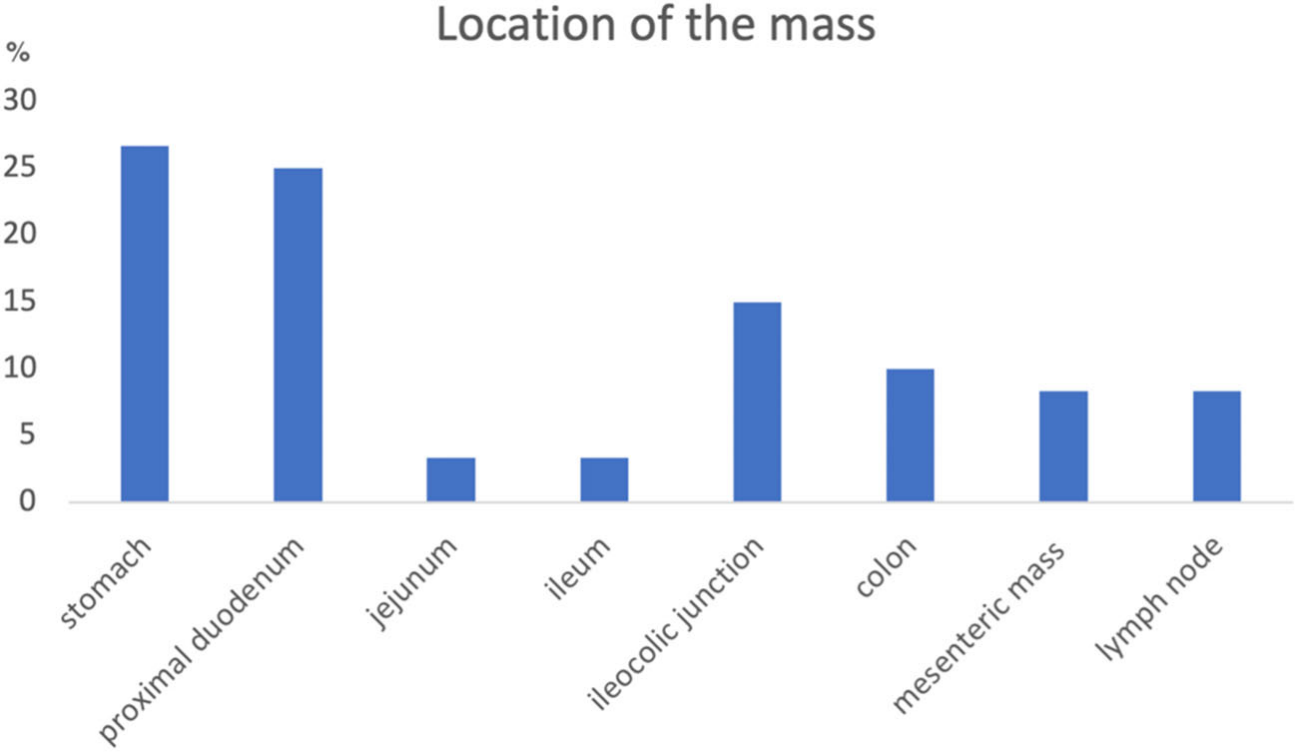
Common locations of the masses found in cats with gastrointestinal sclerosing fibroplasia in this study. Y‐axis represents the % of masses in the location.

The most common location of the masses (Figure 2) was small intestine in 19 (32%) of the 60 cases, including the proximal duodenum 15/60 (25%; Supplementary Figure 1), jejunum 2/60 (3%), ileum 2/60 (3%), and the stomach in 16/60 (27%), followed by the ileocolic junction 9/60 (15%), colon 6/60 (10%), lymph node 5/60 (8%), and mesentery in 5/60 (8%; Supplementary Figure 2). Most of the cats, 51/60 (85%) had only 1 mass; however, in 9/60 (15%) of cats a mass was present in more than 1 location. The additional masses most commonly involved the mesentery and surrounding lymph nodes in 4/9 (44%); in 3 cats the additional masses affected the stomach and proximal duodenum, in 1 cat the mesentery and jejunum, and in 1 cat the proximal duodenum and jejunum. Of note, 1 cat had an eosinophilic skin mass at the same time as GESF. One cat had a mass in the ileocolic junction removed, then presented 7 months later with a mesenteric mass. Another cat had a mass removed from its colon, then presented 2 years later with a pyloric mass, which was also removed, the re‐presented 3 years after that with another pyloric mass. In both of these cases, the cats were not administered corticosteroid therapy until after surgical resection of the second or third mass, respectively and all of the masses in both cats were consistent with GESF on histopathology.

### 3.5 Cytology

Cytology of fine‐needle aspirates (FNA) of the mass was performed in 22 (37%) of the 60 cats and showed eosinophilic inflammation in 10/22 (45%) of cases. In other cases, the cytology was either non diagnostic, or showed necrosis or mixed inflammation. Cytology on FNA of abdominal lymph nodes was performed 22/60 (37%) of cats but was mostly nondiagnostic or showed reactive lymph nodes; eosinophils were only reported in 8/22 (36%) of cases.

### 3.6 Surgery/biopsy

The mass was removed surgically in 22 (37%) of the 60 cats with complete microscopic excision achieved in 18/22 (82%) of the cats. In the remaining 29 (76%) of the 38 cases had a surgical biopsy performed, whereas the diagnosis was achieved on endoscopic biopsies in the other 9 (24%) cats. The cats that had endoscopic biopsies, the mass was located in proximal duodenum in 5/9 (56%) or stomach in 4/9 (44%) of the cases. Surgical complications were reported in 5/22 (22%) of cats, with 3/5 of the cats developing anemia and requiring transfusion (14% of the cats that had surgery to remove or biopsy a GESF mass); all 3 of these cats were anemic on presentation with HTC on presentation being 18, 20 and 26% respectively. One cat developed septic peritonitis requiring a second surgery, 1 cat became anorexic, 1 cat developed chyloabdomen, which has resolved with treatment, and 1 cat developed persistent fecal incontinent after surgical resection of a colonic mass.

### 3.7 Histopathology and culture

**FIGURE 3:**
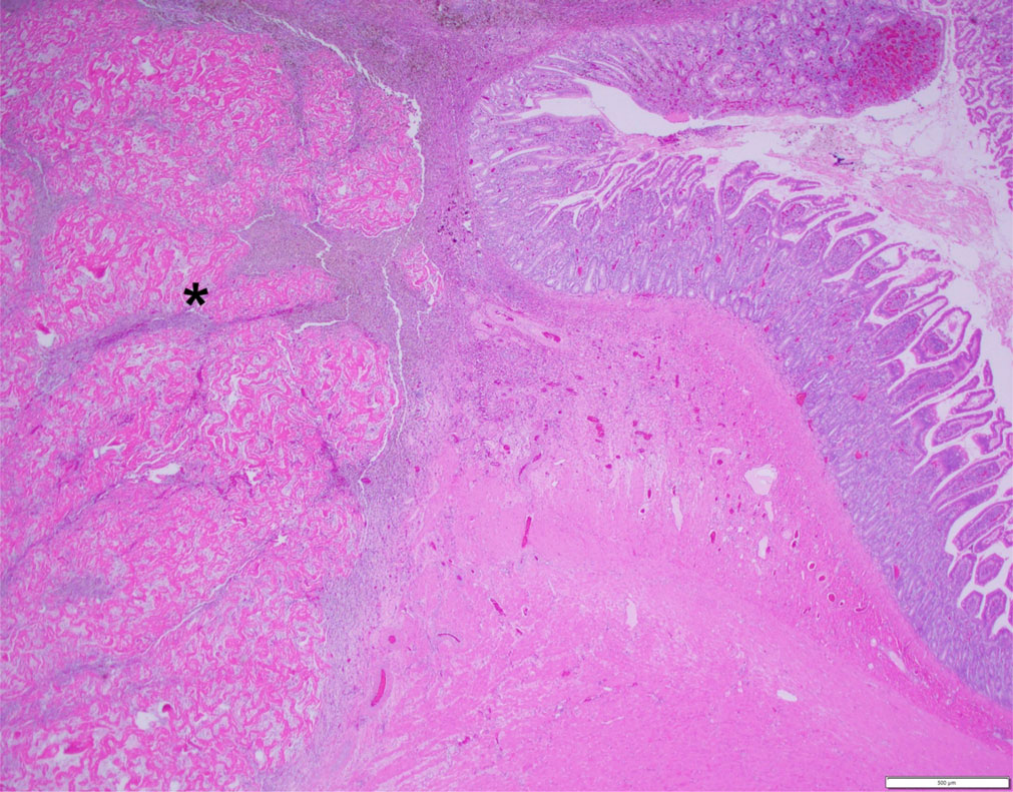
Histological findings of the duodenal mass—the muscularis and submucosa are expanded by a discrete, sparsely cellular mass (*). Hematoxylin & eosin, 20× magnification. Scale bar = 500 μm. Photo credit: Dr Allison Watson from Colorado State University.

**FIGURE 4:**
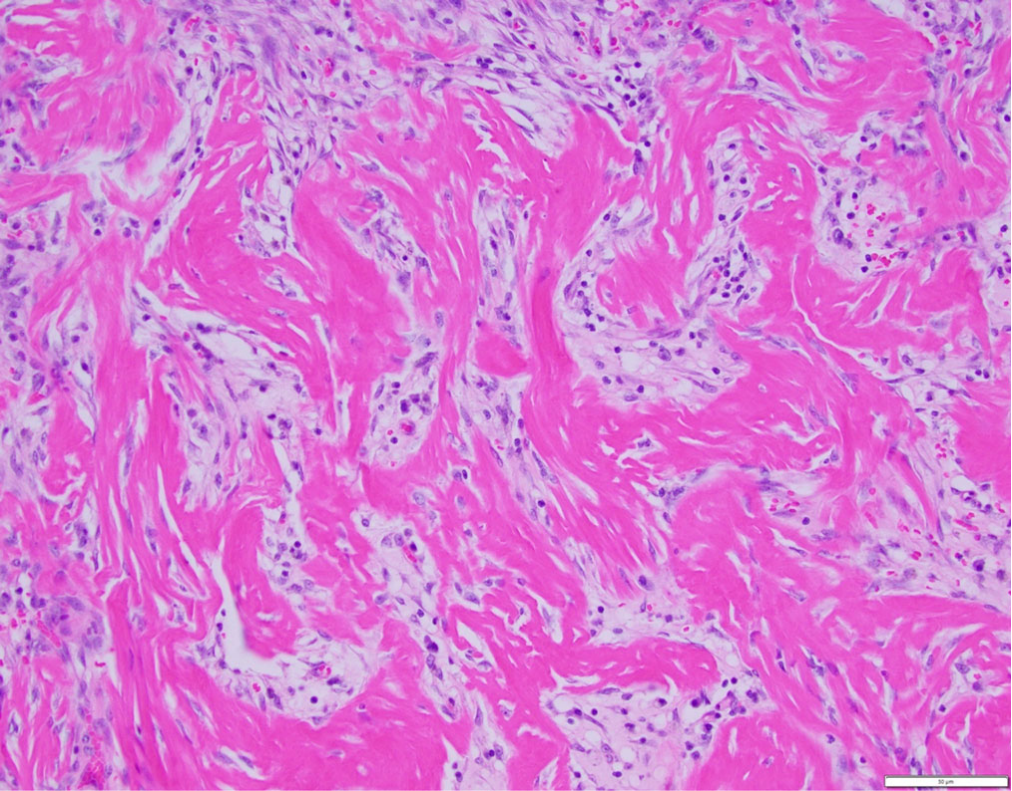
Histological findings of the duodenal mass—the mass is composed of anastomosing trabeculae of sclerotic collagen separated by fibroblasts, macrophages, and small numbers of eosinophils and mast cells. Hematoxylin & eosin, 200× magnification. Scale bar = 50 μm. Photo credit: Dr Allison Watson from Colorado State University.

In all 60 cats, the mass was confirmed as GESF on histopathology (Figures 3 and 4). Of the 22 (37%) out of 60 cases in which the mass was surgically removed, the lesion was completely excised in 13 (59%) cats. In 19/60 (32%) of cats, intralesional bacteria were present on histopathology, and fungal organisms were detected in 1 cat (by positive periodic acid‐Schiff [PAS] staining). Fluorescence in situ hybridization (FISH) was performed in 3 cats and showed *Eubacteria* in 1 cat and *Eubacteria*, *Campylobacter jejuni*, *Salmonella* species and *Escherichia coli* in the second cat and no invasive bacteria in the third cat. Bacterial culture was performed in 18/60 (30%) of cases; 4/18 (22%) were negative, whereas in the others the most common bacteria were *E. coli* (6/12; 50%), *Staphylococcus* species (6/12; 50%), *Enterococcus* species (4/12; 33%) and *Streptococcus* species (1/12; 8%) and *Bacteroides fragilis* (1/12; 8%;). It was not always clear if effusion or mass or swab of the tissue was cultured in some cats. The biopsy that was positive on PAS staining cultured *Candida albicans* as well as *Enterococcus* species and *E. coli*. In 34 (57%) of the 60 cases, additional organs were also biopsied; these included lymph nodes (28/34; 82%), stomach (5/34; 15%), liver (4/34; 12%), duodenum (2/34; 6%), jejunum (1/34; 3%), and omentum (1/34; 3%). In 12 (42%) of the 28 cases where lymph node histopathology was performed cases, results were consistent with GESF, as was the omentum in the 1 case where this site was biopsied.

### 3.8 Treatment

Most cats 59/60 (98%) were treated with corticosteroids, although 1 cat was treated with antibiotics alone. All except for 1 of the cats that were administered corticosteroids were administered prednisolone once daily with a median dose of 1.5 mg/kg/day (IQR, 1.0‐2.0). The median time of cats to be administered corticosteroids was 23 (IQR, 10.5‐49.0) days after first being presented. In 49 cats, the prednisolone dose was changed on with a median of 32 (IQR, 16.0‐60.0) days after starting corticosteroid therapy, and in 13 cats, the prednisolone was discontinued; however, in 11 of these 13 cats, prednisolone had to be restarted at a median time of 114 (IQR, 36.0‐366.0) days after discontinuation because of recurrence of clinical signs. The median time to the lowest dose of prednisolone was 369 (IQR, 195.0‐841.0) days, with a median lowest maintenance dose of 0.65 (IQR, 0.40‐0.90) mg/kg/day needed to control the clinical signs. The most common complications from corticosteroid treatment were hypertriglyceridemia in 5 cats. Hypertriglyceridemia was treated with a low‐fat diet and fish oil in 1 cat, fish oil alone in 1 cat, with low fat diet, fish oil and fenofibrate in 1 cat, and no treatment in the remaining 2 cats, and the development of diabetes mellitus in 3 cats (2 of which went into diabetic remission on insulin therapy—1 of these cats still remain on low dose [0.4 mg/kg/day of prednisolone]). Secondary immunosuppressive agents (cyclosporin or chlorambucil) were prescribed in 14 (23%) of the 60 cats and were discontinued in 8 (57%) of those cats after a media of 80 (IQR, 64.5‐236.3) days.

Antibiotics were prescribed for 43 (72%) of the 60 cats, with the most common antibiotics being penicillins (16/43; 37%), fluoroquinolones (9/43; 26%), metronidazole (6/43; 14%), cephalosporins (5/43; 12%), and clindamycin (2/43; 4%). The average duration of treatment with antibiotics was 34 days (range, 7‐204).

Hydrolyzed or selected protein diets were advised in 37% (22/60) of cats; 41% (9/22) of the owners reported an improvement of clinical signs on hydrolyzed or selected protein diet, although 3 cats would not eat the diet.

### 3.9 Survival

**FIGURE 5:**
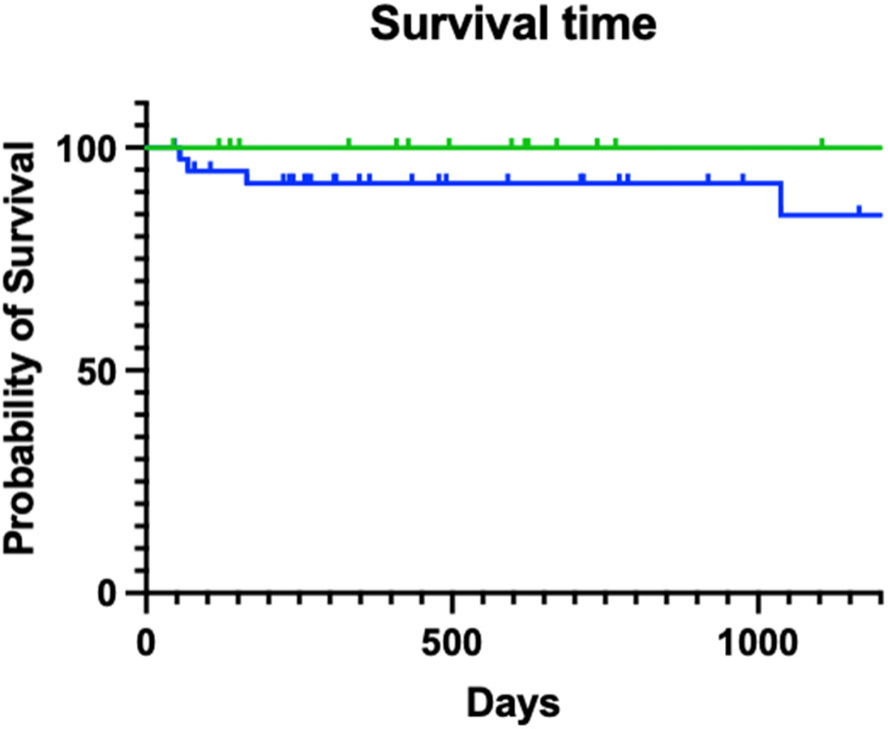
Kaplan‐Meier plot of survival of cats that had a surgical resection of the mass and cats where the mass was biopsied only (*P* = .19). Tick marks represent censored cats. Green line is cats where mass was surgically removed, and blue line is cats where mass was not removed.

As 53 (88%) of the 60 cats still being alive at the time of writing this publication, the median survival time cannot be estimated. Of the 7 cats that died or were euthanized, 4 cats because of poorly controlled GESF, 2 cats of pancreatic neoplasia and 1 cat died of causes unknown. There was no statistical difference between the survival of cats that had a surgical resection of the mass and cats where the mass was biopsied only (*P* = .16; Figure 5). There was no statistical difference between the survival of cats that had a complete resection with clear margins confirmed by histopathology and cats with incomplete resection of the mass and cats where the mass was biopsied only (*P* = .67). There was also no statistical difference between the survival of cats that were treated with corticosteroids only vs secondary immunosuppressive agents (*P* = .41), nor between cats that were treated with antibiotics and cats that were not treated with antibiotics (*P* = .71).

## 4 DISCUSSION

This presents the largest study of GESF in cats to date with cases collected internationally over a 15‐year period. Previous studies had reported the GESF masses in cats to be associated most commonly with the stomach (often near the pylorus) or the intestines, and also affect the abdominal lymph nodes,^1^, ^2^ the mesentery, and retroperitoneum.^3^, ^4^ This is similar to the current study, where the most common location of the GESF masses in cats were the small intestine, stomach, ileocolic junction or colon, whereas in 16% of the cats the mass was associated with lymph nodes or mesentery. In 15% of the cats, GESF masses were present in more than 1 location; in 12 of the cases the local lymph nodes were also affected, and in 1 cat the omentum was involved, showing that this disease can affect a number of locations in each cat. Of note, 1 cat also had an eosinophilic skin mass at the same time as GESF—this has not been reported in cats with GESF; however, subcutaneous masses with eosinophilic infiltration have been reported,^18^ and a recent case report also described possible GESF‐like disease outside the abdominal cavity, in medial retropharyngeal lymph node.^5^

When looking at signalment, the median age of cats with GESF in this study was 5.4 years (range, 1.3‐14.5), which is similar to the reported median age of 7 years, with a range of 2 to 11 years.^2^ Previously, male cats are reported to be more affected by GESF; however, this was not seen in this study.^2^ More than half (58%) of the cats in our study were pedigree cats, with Ragdolls comprising a quarter of the study cats; this is similar to another study that reported Ragdolls to be overrepresented.^2^ Other breeds commonly seen in the current study were Exotic Shorthair (10%) and Persian (8%) cats. It is unclear why pedigree cats appear to be predisposed to develop GESF, notably Ragdolls (25%) and Persian/Exotic cats (18%), and further studies, including genetic analysis, are needed to see if these breeds have a genetic predisposition to develop eosinophilic inflammation as a response to enteric antigens which is the likely cause of GESF. It is important to note that these are also breeds predisposed to feline infectious peritonitis (FIP)^19^; GESF and noneffusive FIP are both differential diagnoses of note for cats presenting with abdominal masses.

In the current study, the median duration of clinical signs was 90 days with most cats showing median of 3 clinical signs, with the most common being weight loss (60%), hyporexia/anorexia (55%), chronic vomiting (37%), lethargy (35%) and chronic diarrhea (27%), which is very similar to a previously reported study; however, they also reported excessive grooming in 50% of cases which was only seen in 2% of the cats in the current study.^2^ Palpation of an abdominal mass is present in 85‐100% of the cats^1^, ^2^; however, this was less common in the current study where a mass was only palpable in 58% of the cats. The prevalence of pyrexia was similar to other studies, 15% vs 18%.^2^

The most common bloodwork abnormality was peripheral eosinophilia, which was present in 50% of the cats in the current study, which is similar to previous studies.^1^, ^2^ Anemia was present in almost third of the cats in the current study, but is not reported. Hypoalbuminemia and hyperglobulinemia were the most common abnormalities, occurring in 27% and 14% of cats, respectively, which is less common than reported in 45% and 67%, respectively.^2^

Large studies evaluating abdominal ultrasonography findings of cats with GESF are lacking to date; however, a study did report 5 cats that had solitary mass with mural thickening and loss of layering in the stomach, duodenum, jejunum, and colon.^16^ In the current study, abdominal ultrasound images were available for review in 50% of the cats, with most cases (83%) showing that the majority of the masses originated from the stomach or intestines. These masses were associated with loss of the intestinal layering and circumferential thickening in most cases, although in 20% there was alteration of the layering rather than loss of it. Enlarged local lymph nodes were present in 90% of the cases, and peritoneal changes in 73%, of which 36% had a peritoneal effusion; however, none of the lesions showed ultrasonographic findings compatible with gastrointestinal perforation.

Intralesional bacteria were identified in 56% of the cases overall (all of the ileocecocolic junction and colon lesions) in 1 study^1^ and in 69% of cats in another study using either culture or conventional light microscopy, special stains and FISH.^2^ In the current study, only 32% of the cases had bacteria present on histopathology, and fungal organisms were detected in 1 cat; however, as 1 limitation of this retrospective study, infectious organisms might have been missed in some cats as FISH was only performed in 3 cats and bacterial culture was performed in only 30% of the cats in this study. Even though bacteria are commonly associated with GESF in cats, fungal organisms are reported once, in a case report of GESF associated with phycomycetes.^20^

The prognosis for cats with GESF is reported as variable, varying from guarded, to cats living for several years.^1^, ^2^, ^16^ Most cats surviving the perioperative period remained well for several years.^2^ In the current study, the median survival time could not be estimated as 88% of the cats still alive at the time of writing this publication. This shows the importance of the correct diagnosis for cats with GESF, as many of these masses can be misdiagnosed as neoplasia, which usually carries a poor prognosis.

Cats being treated with surgery alone have a significantly shorter survival time than those cats treated with surgery and corticosteroids.^1^ Improved survival time is reported when prednisolone was included in the therapeutic regimen, regardless of whether or not cats also had surgery.^2^ In the current study, 98% of the cats were administered corticosteroid therapy, so it is not possible to assess the survival time of the cats with surgery alone; however, there was no statistical difference between the survival of the cats that had their masses surgically resected and cats where their mass were only biopsied including cats with complete resection with clear margins confirmed by histopathology.

Corticosteroids appear to be important in the treatment of cats with GESF. Reoccurrence of masses is reported when surgery was not followed by administration of corticosteroids.^16^, ^17^ In the current study, 1 cat had a mass resected from the ileocolic junction but was not administered corticosteroid therapy, and re‐presented 7 months later with a mesenteric mass. Another cat in this study was diagnosed with GESF in the colon, which was resected, then with a pyloric mass 2 years later, which was also resected, and another pyloric mass 3 years after that; whereas all of these GESF masses were surgically resected, corticosteroid therapy was not administered until after the resection of the third mass. There was no recurrence of abdominal masses in either of these cats after administering corticosteroid therapy for over 1.5 years. The indication to follow surgery with corticosteroid therapy is further supported by 13 cats where prednisolone was discontinued, 85% of these had to have prednisolone restarted a median of 114 days after discontinuation because of recurrence of clinical signs. The median time to the lowest dose of prednisolone was 369 days, with a median lowest maintenance dose of 0.65 mg/kg/day to control the clinical signs; however, as this is a retrospective study, some cats were lost to follow up and it is therefore unclear if prednisolone was tapered further in these cats.

The use of secondary immunosuppressive agents and antibiotics in cats with GESF is reported.^1^, ^2^ In the current study there was no statistical difference between the survival of cats that were treated with corticosteroids only versus including secondary immunosuppressive agents, regardless of whether or not antibiotics were given.

Hydrolyzed or selected protein diets were tried in 37% of the cats in the current study, with 41% of the owners reporting an improvement of clinical signs on these diets. This suggests diet modification as a possible treatment of cats with GESF. However, all of these cats were already being treated with corticosteroids.

## CONFLICT OF INTEREST DECLARATION

Authors declare no conflict of interest.

## OFF‐LABEL ANTIMICROBIAL DECLARATION

Authors declare no off‐label use of antimicrobials.

## INSTITUTIONAL ANIMAL CARE AND USE COMMITTEE (IACUC) OR OTHER APPROVAL DECLARATION

Approved by the University of Edinburgh Ethics Review Committee (VERC Reference: 17.22).

## HUMAN ETHICS APPROVAL DECLARATION

Authors declare human ethics approval was not needed for this study.

## Supporting information


**Data S1.** Supplementary information.
