# Supplementary material for: Clinicopathological findings, treatment, and outcome in 60 cats with gastrointestinal eosinophilic sclerosing fibroplasia
Source: J Vet Intern Med. 2024 Jan 11;38(2):1005–12. doi: 10.1111/jvim.16992 (PMC10937490; doi:10.1111/jvim.16992)
Supplement: Supplementary file 1 — Data S1. Supplementary information. [file JVIM-38-1005-s001.pdf]

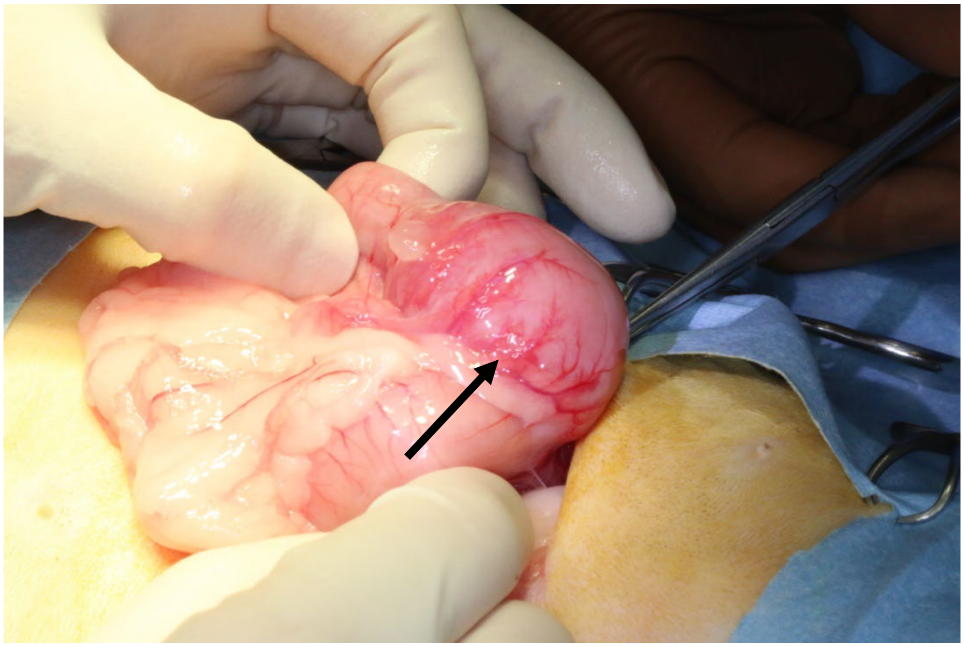

Supplementary Figure 1: Intraoperative photograph of a proximal duodenal mass (arrow) in a cat with gastrointestinal sclerosing fibroplasia. Photo credit: Dr. Atsushi Fujita from Japan Small Animal Medical Center.

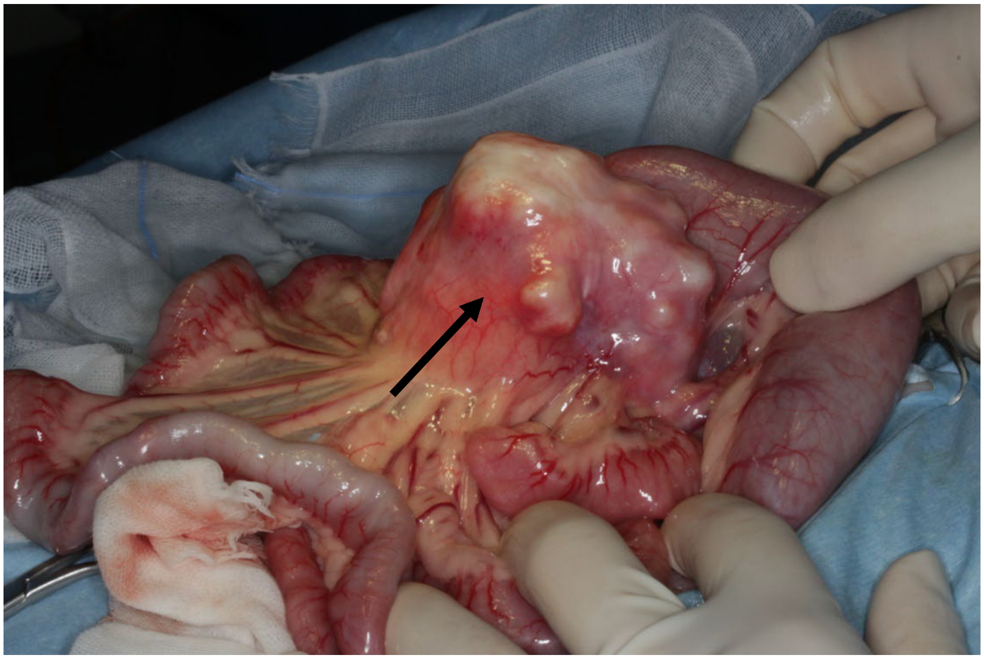

Supplementary Figure 2: Intraoperative photograph of a mesenteric mass (arrow) in a cat with gastrointestinal sclerosing fibroplasia. Photo credit: Dr. Atsushi Fujita from Japan Small Animal Medical Center.
